# Status of maternal serum B vitamins and pregnancy outcomes: New insights from *in vitro* fertilization and embryo transfer (IVF-ET) treatment

**DOI:** 10.3389/fnut.2022.962212

**Published:** 2022-11-11

**Authors:** Ling Zhang, Li-mei Wu, Wei-hai Xu, Yu-qing Tian, Xu-ling Liu, Chen-yun Xia, Lin Zhang, Shi-shi Li, Zhen Jin, Xiang-li Wu, Jing Shu

**Affiliations:** ^1^Center for Reproductive Medicine, Department of Reproductive Endocrinology, Zhejiang Provincial People's Hospital (Affiliated People's Hospital, Hangzhou Medical College), Hangzhou, China; ^2^Department of Postgraduate Education, Jinzhou Medical University, Jinzhou, China; ^3^Key Laboratory of Digital Technology in Medical Diagnostics of Zhejiang Provice, Hangzhou, China; ^4^Calibra Lab, DIAN Diagnostics, Hangzhou, China

**Keywords:** B vitamins, fecundity, *in vitro* fertilization and embryo transfer, preimplantation embryo, pregnancy

## Abstract

The influence of B vitamins on human fertility and infertility treatments remains elusive. Therefore, this study investigated the association of most B vitamins with IVF-ET outcomes. A total of 216 subjects aged <35 year in their first oocyte retrieval cycle were recruited. Blood samples from the participants were collected before the oocyte pick-up procedure, and serum levels of riboflavin, niacin, pantothenic acid, vitamin B6 (including PA and PLP), folate, and methylmalonic acid (MMA) were detected using high-performance liquid chromatography–tandem mass spectrometry (HPLC-MS/MS). Endpoints were classified into three groups according to tertiles (lower, middle, and upper) of each vitamin index, and the association of the serum vitamin status with intermediate and clinical outcomes was analyzed using a generalized estimating equation model. Higher riboflavin levels were associated with elevated probabilities of high-quality embryos, as well as clinical pregnancy after embryo transfer. A greater likelihood of transferable embryos was found in the middle tertile of serum folate. Similarly, a negative correlation of serum MMA, a marker of vitamin B12 deficiency, with high-quality embryos was identified. No significance was observed for other vitamins in terms of all endpoints. Therefore, sufficient levels of pre-conception riboflavin, folate, and vitamin B12 are recommended for successful infertility treatment and pregnancy planning; further evidence is needed to confirm our conclusion.

## Introduction

About 15% couples in China experience conception failure during the first year of pregnancy planning, and about half of these couples seek infertility treatment with assisted reproductive technology (ART), which is usually *in vitro* fertilization and embryo transfer (IVF-ET) (1). However, the prognosis of IVF-ET remains suboptimal, with only around 30% resulting in live birth (1, 2). This highlights the importance of exploring potential modifications to current programs. It is widely accepted that maternal environmental exposure, lifestyle, and nutrition status may substantially influence oocyte quality, a key determinant of preimplantation development and subsequent implantation (3–5). Therefore, an improvement in maternal nutrition before oocyte retrieval is expected to improve IVF-ET outcomes.

B vitamins, as precursors, cofactors, and/or substrates for various biological processes, are crucial for maintaining cellular homeostasis, for example, genomic stability, mitochondrial function, energy metabolism, and redox homeostasis (6–9), aspects that substantially contribute to the quality of oocytes (4, 10). Furthermore, associations between B vitamins and oocyte development potential have been identified, and most studies indicate that supplementation with niacin, folate, pyridoxine, cyanocobalamin, and some bioactive products, such as NMN or NAD+, at critical stages of oocyte development and maturation can protect against oxidative damage and aging and enhance the competence of oocytes and preimplantation embryos (11–15).

Previous studies have attempted to validate the biological relevance of pre-conception B vitamins for female reproduction in couples under infertility treatments, but they have contradictory conclusions. While some show that supplementation with folate and vitamin B12 before oocyte retrieval may improve embryo quality, implantation potential, and live birth during IVF-ET treatment (16–18), others found no beneficial effects on pregnancy outcomes (19, 20), or even reached an opposite association (21). Moreover, all published clinical studies exclusively focus on folate and vitamin B12, the two major vitamins related to the one-carbon metabolism pathway, with no examination of the clinical effects of other B vitamins during infertility treatment, despite their recommendation for general health. Furthermore, additional attention should be paid to the potential adverse effects of B vitamins, including elevated risk of coronary artery disease and fracture (22, 23). Therefore, the relevance of nutritional status of B vitamins to ART outcomes remains open, and more evidence is needed.

In this study, we detected the serum levels of six types of B vitamins, namely, riboflavin, niacin, pantothenic acid, vitamin B6 (PA), vitamin B6 (PLP), and folate, and the nutritional status of vitamin B12 (as indicated by methylmalonic acid) in female partners on the day of the oocyte pick-up (OPU) procedure and analyzed their correlations with the outcomes of IVF-ET treatment using a robust statistical model and a generalized estimating equation (GEE). Our results offer valuable insights into the importance of the optimal B vitamin status during infertility treatment.

## Materials and methods

### Study design and participants

This was a prospective follow-up study conducted in infertile women from September 2017 to December 2019 at the Reproductive Center of Zhejiang Provincial People's Hospital, China. Ethical approval for this study was granted by the Ethics Committee of Zhejiang Provincial People's Hospital (approval code: KT2022018), and all participants signed the written informed consent before participating in the study.

Infertile women who underwent their first IVF-ET cycle were enrolled if they fulfilled the following inclusion criteria: (1) aged <35 year; (2) a serum AMH level >1.10 ng/mL; (3) a gonadotropin-releasing hormone (GnRH) antagonist protocol for controlled ovarian stimulation, and conventional IVF for insemination; (4) a normal range in semen parameters (including sperm forward motility, morphology, sperm density, and DNA fragment) of the partner, as judged by the World Health Organization Laboratory Manual for the Examination and Processing of Human Semen (5th edition, 2010). The exclusion criteria were as follows: (1) poor ovarian response (oocytes retrieved <5); (2) previous chemotherapy or radiotherapy treatment; (3) carrier of an abnormal chromosome karyotype (both balanced and unbalanced ones); (4) diagnosis of female thyroid dysfunction, diabetes, hepatic, or renal disease; (5) uterine cavity abnormalities; (6) familial infertility of any partner; or (7) current smoker or drinker. Finally, a total of 216 patients, who undertook 207 first transfer cycles, were selected.

### Blood sample collection and laboratory assays

From each participant, 3 mL of fasting vein blood was collected in a tube without anticoagulant on the morning of the oocyte pick-up procedure. After clotting, the samples were centrifuged at 1,000 *g* for 15 min, and then the supernatant (serum) was collected, snap-frozen with liquid nitrogen, and stored at 80°C until analysis.

Laboratory assay was conducted at Calibra Lab (DIAN Diagnostics, Hangzhou, China). Sample processing was conducted according to a previous study (24). In brief, 150 μL of serum or quality control sample was added to 350 μL of EVB precipitant for deproteinization, and the deproteinized sample was mixed on ice at 1,500 rpm for 10 min. Then, the mixture was centrifuged at 15,000 rpm at 4°C for 5 min, and 100 μL of supernatant was gently transferred to a new vial on ice. Then, the supernatant was injected into a Calibra C18 column of 2.1 × 100 mm (2.5 μm) and mounted in a thermostatic column compartment set at 40°C. The mobile phase of the binary solvent system consisted of 10 mM ammonium acetate (eluent A) and acetonitrile solution containing 0.1% formic acid (eluent B). MS detection was performed with an AB 4,500 MD tandem mass spectrometer in electrospray ionization (ESI) and multiple reaction monitoring mode. Supplementary Table S1 shows the mass-to-charge ratio (m/z) of the precursor and product ions, and fragmentor voltage and collision energy values were determined for each vitamin. All data were analyzed by SCIEX Analyst software v.1.6.2 (AB Sciex LLC, Framingham, MA, USA). The lower limits of quantification (LLOQ) of each analyte were as follows: riboflavin 1.00 pg/mL, niacin 2.11 pg/mL, pantothenic acid 5.27 pg/mL, 4-pyridoxic acid 1.00 pg/mL, pyridoxal-5'-phosphate (PLP) 2.00 pg/mL, 5-methyltetrahydrofolic acid 2.00 pg/mL, and methylmalonic acid (MMA) 4.30 pg/mL.

### IVF-ET procedure

Controlled ovarian stimulation was performed using GnRH antagonist protocols, as described previously (25), and gonadotropins (Gn) were administrated in a flexible way from cycle day 2 at a dose of 100–300 U per day. When the diameter of leading follicles reached 12 mm, a daily dose of 0.25 mg GnRH antagonist was injected subcutaneously. Final oocyte maturation was triggered by recombined hCG, GnRH agonist, or their combination. Cumulus–oocyte complexes (COCs) were aspirated from large follicles (diameter ≥16 mm) by transvaginal puncture under ultrasound guidance at 36 h after the oocyte maturation trigger. Retrieved oocytes were inseminated by conventional IVF 39–41 h after trigger. Fertilization was checked 16–18 h after insemination, with normal fertilization defined as zygotes with two pronuclei (2 PN). Zygotes were transferred into Sydney IVF Cleavage Medium (CM, COOK MEDICAL, Australia) for further culture. The quality of embryos at day 3 was evaluated based on blastomere symmetry and fragmentation, as described previously (good, fair, and poor) (26). Day 3 embryos graded as “good” with a blastomere count ≥7 were defined as high-quality embryos, and those graded over “fair” with a blastomere count ≥4 were defined as transferable embryos. The high-quality embryos were directly cryopreserved by vitrification, while the remainder underwent a continuous blastocyst culture to day 5 or day 6 and were scored according to the Gardner score system (27). Only transferable blastocysts with inner cell mass or trophectoderm graded above “B” were cryopreserved. All vitrification and warming procedures were conducted according to the manufacturer's instructions (Vitrolife, Göteborg, Sweden). A frozen-all strategy was used for all IVF-ET treatment cycles.

For endometrial preparation during frozen embryo transfer, a natural, hormone replacement (HRT), or induced ovulation cycle was used. A maximum of two embryos were thawed and transferred under ultrasound guidance. Clinical pregnancy was defined as visualization of gestational sac/s under ultrasound examination 5 weeks post-transfer. Once pregnancy was achieved, progesterone and estradiol supplementation were continued until 10 weeks of gestation. Endpoints of the present study included clinical pregnancy rate, live birth rate, implantation rate (defined as the ratio of gestation sacs on ultrasound to the number of embryos transferred), early miscarriage (defined as spontaneous loss of a clinical pregnancy of <12 weeks of gestation) rate, 2PN fertilization rate, transferable embryo rate, and high-quality embryo rate.

### Assessment of covariates

Factors that might influence the outcome of infertility treatment were considered covariates, including anthropometric data, baseline clinical characteristics, and treatment parameters during IVF-ET. Anthropometric data included female age and body mass index (BMI, kg/m^2^); baseline clinical characteristics included infertility duration, primary infertility, infertility diagnosis (categorized as endometriosis, ovulation disorders, tubal factor, and unexplained infertility), and measures of ovarian reserve such as serum anti-Mullerian hormone (AMH), basic follicle-stimulating hormone (basic FSH), and antral follicle count (AFC); and treatment parameters included total doses and days of gonadotropin (Gn) and trigger method (categorized as hCG, GnRH-a, or dual trigger).

For clinical outcomes after transfer, additional covariates were assessed, including the number of transferred embryos (1 or 2), embryo stages (cleavage or blastocyst stage), and endometrial thickness at transfer day. All information on the covariates was obtained *via* clinical examination and consultation, and recorded in an infertility case management system.

### Statistical analysis

Data were analyzed by SPSS statistical software (v.21.0, IBM Corp., USA). The distribution of each continuous variable was assessed by using the Kolmogorov–Smirnov test. Due to non-normal distribution (Supplementary Table S7), all the continuous variables of this study were expressed as median (interquartile range, IQR), and the categorical variables were expressed as *n* (%). The association between various B vitamins, serum B vitamins, and baseline indices of ovarian reserve were analyzed using Spearman rank correlations, with the coefficient *r*_*s*_ as the strength of association. Given that no recommended threshold values for serum B vitamin indices are available for women with infertility, included cycles were assigned into tertiles (categorized as lower, middle, and high) by each of the B vitamin indices. Comparisons among the tertiles were performed using the non-parametric Kruskal–Wallis test and Fisher's exact test for continuous and categorical variables, respectively. Contribution of B vitamins to study endpoints were calculated using generalized estimating equation (GEE) models adjusting for certain covariates. For intermediate outcomes (including 2PN fertilization, transferable embryo, and high-quality embryo) and implantation, a Poisson distribution with log link function was chosen, whereas for clinical pregnancy, live birth, and early miscarriage, a binomial distribution with log link function was used. The offset for Poisson models was specified as the logarithm of the number of oocytes or transferred embryos. Covariates that showed a univariate relationship with outcome or those considered clinically relevant were brought into the multivariate analyses. Correlation of various vitamin indices were calculated as an adjusted OR value, with the lower tertile serving as the reference. A two-tailed *P* < 0.05 was set for statistical significance.

## Results

### Included cycles

All the participants included were of Han ethnicity. The median age of female partners was 30 (IQR: 28–32) year, median BMI was 21.10 (IQR: 19.80–23.20) kg/m^2^, and median AMH was 3.92 (IQR: 3.01–5.48) ng/mL. Other baseline characteristics of female partners are summarized in Table 1. For 216 oocyte pick-up cycles, a total of 2,643 oocytes were retrieved (2,337 mature oocytes), yielding an average of 11.40 ± 4.45 oocytes per cycle. After *in vitro* insemination and culture, an average of 7.61 ± 3.57 2PN zygotes (70.61%), 6.94 ± 3.50 day 3 transferable cleavage embryos (64.42%), and 4.60 ± 3.01 high-quality embryos (42.72%) per cycle were obtained. Of the 216 couples included, nine couples did not undertake embryo transfer due to the lack of transferable embryos (four couples) or delayed fertility intention (five couples). Finally, 207 couples undertook at least one embryo transfer cycle, and their first cycles were extracted for analysis of clinical outcomes after embryo transfer. These 207 cycles resulted in a total implantation rate of 49.28% (170/345), a clinical pregnancy rate of 63.29% (131/207), a live birth rate of 56.04% (116/207), and an early miscarriage rate of 11.45% (15/131).

**Table 1:** Demographic characteristics and serum B vitamins of included couples.

**Parameter**	**Values**
No. of couples (*n*)	216
Female age (year)	30 (27–32)
BMI (Kg/m^2^)	21.10 (19.80–23.20)
AMH (ng/mL)	3.92 (3.01–5.48)
Basic FSH (IU/L)	5.18 (4.50–6.01)
Antral follicle count (AFC, *n*)	14 (11–17)
Infertility duration (year)	2 (1–3)
Primary infertility, *n* (%)	102 (47.20)
Endometriosis, *n* (%)	29 (13.43)
Tubal factor, *n* (%)	38 (17.59)
Ovulation disorder, *n* (%)	44 (20.37)
Unexplained, *n* (%)	16 (7.41)
Riboflavin (ng/mL)	9.48 (5.79–15.10)
Niacin (ng/mL)	8.35 (4.12–14.53)
Pantothenic acid (ng/mL)	32.63 (26.71–40.26)
Vitamin B6 (PA, ng/mL)	1.79 (1.36–2.83)
Vitamin B6 (PLP, ng/mL)	8.84 (6.59–14.81)
Folate (ng/mL)	17.02 (10.25–27.80)
MMA (ng/mL)	10.63 (7.65–14.84)

Quantitative data were described as median [interquartile range (IQR)]. MMA, methylmalonic acid.

### Distribution of serum B vitamin status and their correlation with indices of ovarian reserve

The distribution of serum B vitamins is given in Table 1. By the criteria of Mayo Clinic, Labcorp, or Quest (Supplementary Table S2), all six vitamins presented considerable serum loads, with the highest absolute value for pantothenic acid, and the lowest value for vitamin B6-PA. For most patients, serum methylmalonic acid (MMA) was in the normal range. Considering biological relevance, we combined vitamin B6-PA and vitamin B6-PLP as vitamin B6 for further statistical analysis and discussion. Of these vitamins, riboflavin, pantothenic acid, vitamin B6, and folate showed significant positive correlations, whereas niacin and MMA were relatively independent of others, except for a weak correlation between folate and MMA (Table 2). There was no significant correlation between the serum status of all vitamin indices and ovarian reserve function (basic FSH, AFC, and AMH), except for MMA. The serum MMA level showed a weak negative relationship with the AMH level (*rs* = −0.136, *p* = 0.046, Table 2).

**Table 2:** Association between various B vitamins, and between serum B vitamin status and ovarian reserve (*r*_*s*_).

	**Riboflavin**	**Niacin**	**Pantothenic acid**	**Vitamin B6**	**Folate**	**MMA**
Niacin	0.115^*^					
Pantothenic acid	0.243^**^	0.099				
Vitamin B6	0.340^**^	0.104	0.442^**^			
Folate	0.140^*^	−0.010	0.403^**^	0.462^**^		
MMA	−0.069	0.051	−0.055	0.075	−0.166^*^	
Basic FSH	−0.071	0.049	0.001	−0.011	−0.089	−0.005
AFC	−0.091	0.034	−0.037	−0.042	−0.034	0.009
AMH	0.006	0.077	0.018	−0.033	−0.003	−0.136^*^

Correlation between serum B vitamin status and reproductive hormones was estimated by the coefficient r_s_ with the Spearman rank correlation (

* P < 0.05,

** P < 0.01). Vitamin B6 contained two members, pyridoxic acid (PA) and 5'-phosphates pyridoxal (PLP); MMA, methylmalonic acid; AMH, anti-Mullerian hormone; AFC, antral follicle count; coefficient r_s_.

### Maternal serum status of B vitamins and intermediate outcomes

The enrolled cycles were divided into three groups according to the serum concentration of each B vitamin. Basic characteristics and parameters mentioned before were compared among the tertiles of each vitamin index, which showed a considerable balance for most comparisons (Supplementary Table S3). The univariate association of theses covariates was calculated and is described in Supplementary Table S5.

The rates of 2PN zygotes, transferable, and high-quality embryos were summarized into three groups according to the tertiles of each vitamin index (Table 3). With the increase in serum riboflavin, the probability for transferable embryos increased from 57.95% in the lower tertile, to 61.72% in the middle tertile, to 63.20% in the upper tertile; the high-quality embryo rate ascended from 36.56% in the lower tertile to 42.94 and 41.75% in the middle and upper tertiles, respectively. After adjusting for age, BMI, infertility duration, and days of Gn, the advantage in high-quality embryos remained for the middle group over the lower tertile [OR = 1.21 (95% CI: 1.04–1.41), *P* = 0.013]. Similarly, we also revealed a substantial advantage for folate step-up, with the transferable embryo rates rising from 56.97% in the lower tertile to 63.27 and 62.44% in the middle and upper tertiles, respectively (*P* = 0.020), but only a marginally remarkable OR remained for the middle tertile after adjustment [1.13 (95% CI: 1.00–1.28) (*P* = 0.051)]. A negative correlation of the serum MMA level with high-quality embryos was identified, with the probabilities decreasing from 43.01% in the lower tertile to 41.54 and 36.44% for the middle and upper tertiles (*P* = 0.020). Correspondingly, multivariate GEE analysis still exhibited a substantial inferiority for the upper tertile to the lower tertile after adjusting for covariates [OR = 0.81 (95% CI: 0.69–0.95), *P* = 0.008]. This result firmly indicated that the vitamin B12 status is associated with embryo quality. Furthermore, a higher serum pantothenic acid level was associated with lower 2PN fertilization in Fisher's exact test, but the significance did not remain in GEE–Poisson analysis with or without adjusting covariates (Supplementary Table S5 and Table 3). No significant correlation was found between serum levels of other B vitamins and intermediate outcomes (Table 3).

**Table 3:** Associations between serum B vitamin concentrations and intermediate IVF outcomes.

**Indices of B vitamins**	**Content level (ng/mL)**	**Cycles (*n*)**	**2 PN zygotes**		**Day3 transferable embryos**		**Day3 high-quality embryos**	
			***n*/*N* (%)**	**aORs (95% CI)^‡^**	***n*/*N* (%)**	**aORs (95% CI)**	***n*/*N* (%)**	**aORs (95% CI)**
Riboflavin	T1 (1.49–6.70)	72	551/837 (65.83)	Reference	485/837 (57.95)	Reference	306/837 (36.56)	Reference
	T2 (6.71–12.64)	72	557/838 (66.63)	1.04 (0.92–1.17)	516/838 (61.72)	1.10 (0.97–1.24)	359/838 (42.94)	**1.21 (1.04–1.41)**
	T3 (12.83–74.84)	72	535/788 (67.89)	1.05 (0.93–1.18)	498/788 (63.20)	1.11 (0.97–1.26)	329/788 (41.75)	1.14 (0.98–1.34)
	*P*-value (*Q*-value)^†^		0.667 (0.848)		0.083 (0.249)		0.020 (0.120)	
Niacin	T1 (1.41–5.26)	72	549/827 (66.46)	Reference	502/827 (60.77)	Reference	338/827 (40.92)	Reference
	T2 (5.32–11.98)	72	561/829 (67.75)	1.00 (0.89–1.13)	509/829 (61.47)	0.99 (0.87–1.12)	322/829 (38.89)	0.92 (0.79–1.07)
	T3 (12.04–42.61)	72	533/807 (66.05)	0.97 (0.86–1.10)	488/807 (60.47)	0.97 (0.85–1.10)	334/807 (41.39)	0.97 (0.84–1.13)
	*P*-value (*Q*-value)^†^		0.762 (0.848)		0.926 (0.948)		0.539 (0.647)	
Pantothenic acid	T1 (16.30–28.87)	71	578/816 (70.83)	Reference	510/816 (62.50)	Reference	332/816 (40.69)	Reference
	T2 (29.03–36.61)	73	569/860 (66.16)	0.94 (0.83–1.06)	526/860 (61.16)	0.98 (0.86–1.11)	353/860 (41.05)	0.98 (0.84–1.14)
	T3 (36.86–154.92)	72	496/787 (63.18)	0.91 (0.80–1.03)	463/787 (58.98)	0.97 (0.85–1.10)	309/787 (39.36)	0.99 (0.84–1.16)
	*P*-value (*Q*-value)^†^		0.004 (0.024)		0.314 (0.628)		0.742 (0.742)	
Vitamin B6	T1 (4.24–8.66)	72	505/755 (66.98)	Reference	463/755 (61.41)	Reference	319/755 (42.31)	Reference
	T2 (8.77–13.84)	72	616/897 (68.67)	1.04 (0.92–1.17)	545/897 (60.76)	1.00 (0.88–1.13)	338/897 (37.68)	0.89 (0.76–1.04)
	T3 (13.94–111.30)	72	522/811 (64.44)	0.98 (0.86–1.11)	491/811 (60.62)	1.01 (0.89–1.15)	337/811 (41.60)	1.01 (0.87–1.18)
	*P*-value (*Q*-value)^†^		0.167 (0.501)		0.948 (0.948)		0.118 (0.236)	
Folate	T1 (1.41–12.10)	72	529/818 (64.67)	Reference	466/818 (56.97)	Reference	308/818 (37.65)	Reference
	T2 (12.13–24.36)	72	580/856 (67.84)	1.07 (0.95–1.20)	541/856 (63.27)	**1.13 (1.00–1.28)**	355/856 (41.52)	1.12 (0.96–1.30)
	T3 (24.61–54.51)	72	534/789 (67.77)	1.06 (0.93–1.19)	492/789 (62.44)	1.11 (0.98–1.26)	331/789 (42.01)	1.14 (0.98–1.34)
	*P*-value (*Q*-value)^†^		0.318 (0.636)		0.019 (0.114)		0.153 (0.236)	
MMA	T1 (2.83–8.36)	72	583/865 (67.40)	Reference	538/865 (62.20)	Reference	372/865 (43.01)	Reference
	T2 (8.40–12.75)	72	530/792 (66.92)	0.98 (0.86–1.10)	486/792 (61.36)	0.96 (0.85–1.09)	329/792 (41.54)	0.93 (0.80–1.08)
	T3 (12.83–59.53)	72	530/802 (65.92)	0.95 (0.84–1.07)	475/802 (59.08)	0.92 (0.81–1.04)	293/802 (36.44)	**0.81 (0.69–0.95)**
	*P*-value (*Q*-value)^†^		0.848 (0.848)		0.444 (0.666)		0.020 (0.060)	

2 PN refers to two pronuclei.

† Fisher's exact test for categorical variables were conducted. Multiple testing P-values were corrected as Q values using the Benjamini–Hochberg procedure.

‡ Effect of the serum B vitamin level on intermediate IVF outcomes was evaluated as odd ratios (95% CI) with the lowest tertile as reference. The Poisson model with a log link function was used. aORs: OR values adjusted for age, BMI, infertility duration, and days of Gn.

Bold values means P < 0.05.

To deal with the problem with multiple testing, univariate *P*-values were also corrected as *Q*-values using the Benjamini–Hochberg procedure. In this conservative way, most of the previous relevance became null, except for that of pantothenic acid on 2PN fertilization (Table 3).

### Maternal serum B vitamin status and clinical outcome after embryo transfer

The recruited frozen embryo transfer cycles were divided according to the tertiles of serum for each B indices. Comparison in the covariates among the tertiles and their univariate association are shown in Supplementary Tables S4, S6 respectively. The amount of embryos transferred showed a significant univariate association in terms of clinical pregnancy and live birth (Supplementary Table S6).

The clinical outcomes after embryo transfer including implantation, clinical pregnancy, live birth, and early miscarriage are described in Table 4. There was a significant difference in implantation and clinical pregnancy among the tertiles of serum riboflavin. The implantation rate increased from 39.13% in the lower tertile to 56.36 and 52.50% in the middle and upper tertiles, respectively (*P* = 0.024), while the clinical pregnancy rate increased from 49.30 to 68.12 and 73.13%, respectively (*P* = 0.009, Table 4). Similarly, this relevance did not exist yet after Benjamini–Hochberg correction (Table 4).

**Table 4:** Associations between serum levels of B vitamins and the clinical outcomes after embryo transfer.

**Indices of B vitamins**	**Content level (ng/mL)**	**Cycles (*n*)**	**Implantation**		**Clinical pregnancy**		**Live birth**		**Early miscarriage**	
			***n*/*N* (%)**	**aORs (95% CI)^‡^**	***n*/*N* (%)**	**aORs (95% CI)**	***n*/*N* (%)**	**aORs (95% CI)**	***n*/*N* (%)**	**aORs (95% CI)**
Riboflavin	T1 (1.49–6.70)	71	45/115 (39.13)	Reference	35/71 (49.30)	Reference	33/71 (46.48)	Reference	2/35 (5.71)	Reference
	T2 (6.71–12.64)	69	62/110 (56.36)	1.44 (0.98–2.12)	47/69 (68.12)	2.45 (1.18–5.10)	39/69 (56.52)	1.58 (0.79–3.17)	8/47 (17.02)	3.31 (0.63–17.31)
	T3 (12.83–74.84)	67	63/120 (52.50)	1.33 (0.91–1.96)	49/67 (73.13)	2.49 (1.17–5.29)	44/67 (65.67)	1.92 (0.94–3.94)	5/49 (10.20)	2.08 (0.36–12.12)
	*P*-value (*Q*-value)^†^		0.024 (0.144)		0.009 (0.054)		0.076 (0.456)		0.266 (0.837)	
Niacin	T1 (1.41–5.26)	69	55/115 (47.83)	Reference	43/69 (62.32)	Reference	36/69 (52.17)	Reference	7/43 (16.28)	Reference
	T2 (5.32–11.98)	67	58/111 (52.25)	1.10 (0.76–1.60)	47/67 (70.15)	1.56 (0.73–3.32)	43/67 (64.18)	1.74 (0.85–3.58)	4/47 (8.51)	0.50 (0.13–1.94)
	T3 (12.04–42.61)	71	57/119 (47.90)	0.99 (0.68–1.44)	41/71 (57.75)	0.80 (0.39–1.64)	37/71 (52.11)	0.96 (0.48–1.92)	4/41 (9.76)	0.58 (0.15–2.23)
	*P*-value (*Q*-value)^†^		0.748 (0.748)		0.313 (0.695)		0.264 (0.792)		0.471 (0.837)	
Pantothenic acid	T1 (16.30–28.87)	69	63/118 (53.39)	Reference	46/69 (66.67)	Reference	40/69 (57.97)	Reference	6/46 (13.04)	Reference
	T2 (29.03–36.61)	72	57/119 (47.90)	0.88 (0.61–1.26)	44/72 (61.11)	0.82 (0.39–1.71)	41/72 (56.94)	1.01 (0.50–2.05)	3/44 (6.82)	0.52 (0.12–2.28)
	T3 (37.00–124.64)	66	50/108 (46.30)	0.88 (0.60–1.28)	41/66 (62.12)	0.92 (0.44–1.92)	35/66 (53.03)	0.90 (0.44–1.82)	6/41 (14.63)	1.31 (0.38–4.57)
	*P*-value (*Q*-value)^†^		0.529 (0.728)		0.769 (0.823)		0.831 (0.940)		0.483 (0.837)	
Vitamin B6	T1 (3.53–7.59)	69	62/117 (52.99)	Reference	47/69 (68.12)	Reference	40/69 (57.97)	Reference	7/47 (14.89)	Reference
	T2 (7.86–12.94)	69	55/115 (47.83)	1.10 (0.76–1.59)	40/69 (57.97)	1.10 (0.52–2.30)	37/69 (53.62)	0.97 (0.48–1.96)	3/40 (7.50)	1.33 (0.37–4.75)
	T3 (13.13–88.44)	69	53/113 (46.90)	1.02 (0.69–1.51)	44/69 (63.77)	0.68 (0.32–1.43)	39/69 (56.52)	0.83 (0.40–1.71)	5/44 (11.36)	0.48 (0.10–2.36)
	*P*-value (*Q*-value)^†^		0.607 (0.728)		0.463 (0.695)		0.872 (0.940)		0.558 (0.837)	
Folate	T1 (1.410–12.10)	68	55/115 (47.83)	Reference	41/68 (60.29)	Reference	37/68 (54.41)	Reference	4/41 (9.76)	Reference
	T2 (12.13–24.36)	70	64/120 (53.33)	1.12 (0.78–1.62)	49/70 (70.00)	1.66 (0.78–3.51)	42/70 (60.00)	1.37 (0.67–2.79)	7/49 (14.29)	1.53 (0.41–5.77)
	T3 (24.61–54.51)	69	51/110 (46.36)	0.99 (0.67–1.47)	41/69 (59.42)	1.16 (0.56–2.41)	37/69 (53.62)	1.12 (0.55–2.29)	4/41 (9.76)	1.17 (0.25–5.37)
	*P*-value (*Q*-value)^†^		0.533 (0.728)		0.356 (0.695)		0.711 (0.940)		0.733 (0.880)	
MMA	T1 (2.83–8.36)	70	63/122 (51.64)	Reference	46/71 (64.79)	Reference	40/71 (56.34)	Reference	6/46 (13.04)	Reference
	T2 (8.44–12.75)	69	51/113 (45.13)	0.88 (0.61–1.28)	41/68 (60.29)	0.90 (0.43–1.85)	37/68 (54.41)	0.98 (0.49–1.97)	4/41 (9.76)	0.88 (0.22–3.51)
	T3 (12.83–59.53)	67	56/110 (50.91)	0.99 (0.69–1.43)	44/68 (64.71)	1.20 (0.57–2.52)	39/68 (57.35)	1.22 (0.60–2.48)	5/44 (11.36)	0.92 (0.24–3.61)
	*P*-value (*Q*-value)^†^		0.558 (0.728)		0.823 (0.823)		0.940 (0.940)		0.891 (0.891)	

† Fisher's exact test for categorical variables were conducted. Multiple testing P-values were corrected as Q-values using the Benjamini–Hochberg procedure.

‡ Effect of the serum B vitamin level on clinical outcomes including implantation, clinical pregnancy, live birth, and early miscarriage was evaluated as odd ratios (95% CI) with the lowest tertile as reference. GEE log binomial models were specified for clinical pregnancy, live birth, and early miscarriage, while a GEE Poisson model was used for implantation. aORs: OR values adjusted for covariates. For analysis on clinical pregnancy and live birth, age, BMI, primary infertility, infertility duration, embryo stage (cleavage or blastocyst), number of transferred embryos were adjusted, for implantation, age, BMI, primary infertility, infertility duration and embryo stage (cleavage or blastocyst) were adjusted, and for early miscarriage, age, BMI, primary infertility and infertility duration were adjusted.

Bold values means P < 0.05.

After adjusting for clinically relevant covariates, ORs for clinical pregnancy were 2.45 (95% CI: 1.18–5.10, *P* = 0.016) and 2.49 (95% CI: 1.17–5.29, *P* = 0.018) for the middle and upper tertiles, respectively. However, the ORs for implantation were not significant. None of the other B vitamin indices presented any significant association with the likelihood of implantation, clinical pregnancy, live birth, or early miscarriage (Table 4).

## Discussion

It is understood that a balanced diet is critical to the success of infertility treatment. However, evidence supporting such nutritional advice is lacking. The potential importance of B vitamins in human fertility has been well studied (28), but little evidence exists for their relevance regarding oocyte competence, early embryo development, and pregnancy establishment. Excepting the one-carbon-related members, folate, and vitamin B12, the influence of other B vitamins on fertility and infertility treatments remains to be understood.

To our knowledge, this prospective study is the first to assess the pre-conception serum status of most B vitamins using high-performance liquid chromatography–tandem mass spectrometry (HPLC-MS/MS) in the infertility clinic and, importantly, to estimate their correlations with the outcomes of IVF-ET treatment. Our data demonstrated significant associations of higher riboflavin and folate levels with improved oocyte competence and embryo quality, and a negative correlation of vitamin B12 deficiency with the likelihood of high-quality embryos. These results provide a novel and more comprehensive insight into the biological implication of B vitamins in the maintenance of oocyte quality and female fertility.

Cytoplasmic bioenergetic capacity and mitochondrial ATP generation are directly related to oocyte competence and embryo development (29, 30), and undermined metabolic activity may result in the decreased developmental potential of oocytes and embryos (31, 32). However, the causal influence of metabolism-related vitamins such as thiamine, riboflavin, and niacin on female fecundity has not yet been reported. Bioactive derivatives of riboflavin and niacin including flavin adenine dinucleotide (FAD), flavin mononucleotide (FMN), and nicotinamide adenine dinucleotide (NAD+) are universally implicated in the maintenance of mitochondrial function and energy homeostasis (33, 34). Previous evidence demonstrates that the elimination of influx of riboflavin *via* immuno-blocking of riboflavin carrier protein in primate ovulated oocytes can cause early embryo degeneration and that lower intracellular flavins or FAD+ levels are associated with an age-related decline of oocyte quality or chromosome instability in human preimplantation embryos (35–37). This evidence suggests that riboflavin deficiency may impair oocyte quality and embryo development. For the first time in the present study, riboflavin was significantly linked to intermediate outcomes and clinical pregnancy during IVF-ET treatment, and the probability of producing high-quality embryos increased by 21% in the middle tertile of riboflavin, and clinical pregnancy increased by 145 and 149% for middle and upper tertiles, respectively. Combined with this and other evidence, the riboflavin status might be an important component for oocyte quality.

No association of niacin was found with the developmental competence of oocytes in this study. Much evidence indicates that NAD+ is implicated in various key biochemical processes, such as NAD+-dependent deacylation and ADP ribosylation, and it is essential for maintaining oocyte quality and female fertility (12, 38). This inconsistency might be explained by the pattern of NAD+ biosynthesis pathways. In mammals, NAD+ is largely generated through the salvage pathway, during which synthetic substrate nicotinamide (NAM) is mostly derived from the by-products of NAD+-consuming enzymes or dietary tryptophan. Nicotinamide phosphoribosyl transferase (NAMPT)-catalyzed NAM transformation is the rate-limiting reaction for intracellular NAD+ biosynthesis (12), and the intracellular NAD+ pool mainly depends on the capacity of NAMPT, instead of the level of niacin supply under conditions of sufficient dietary tryptophan or niacin (39). Thus, it is not surprising that serum niacin does not show any relation to the outcomes of IVF-ET treatment as intracellular NAD+ levels may remain constant and independent, if serum niacin is sufficient under the fortified status.

The importance of the one-carbon metabolism pathway in the maintenance of human fertility has been discussed for decades, and the mainstream belief is that the nutritional status of folic acid and vitamin B12 is essential for successful conception, birth defect prevention, and promising reproductive outcomes after infertility treatment (40, 41). Consistently, in the present study, we found a negative association of serum markers of vitamin B12 deficiency with preimplantation embryo development, with a decrement of 19% of high-quality embryo in the upper tertile (12.83–59.53 ng/mL). In addition, a marginal significant increase in transferable embryos was also observed under the folate middle tertile (12.13–24.36 ng/mL). The mechanisms behind these associations remain elusive. Some believe that the requirement for one-carbon metabolism is enhanced during gametogenesis, folliculogenesis, and early embryo development because of rapid cell division, intensive intracellular DNA synthesis, and methylation/demethylation modification (42). Moreover, attention should be paid to the inverse *U*-shaped dose–response curve for vitamin B12 and folate in aspects of fertility, such that each has a Goldilocks zone. This putative feature has been supported by a recent animal study, which demonstrates that while 200 pM of cyanocobalamin supplement during *in vitro* oocyte maturation results in a higher likelihood of blastulation, and lower preimplantation development arrest and degeneration, doses as high as 300 or 500 pM undermine embryo developmental potential and increase the probability of degeneration (11). For folate, although most researchers demonstrated a positive correlation of the serum folate level with reproductive outcomes (17, 18, 43), some showed an impaired outcome if the serum folate level is higher than 33.0 ng/mL (21). These biphasic patterns for vitamin B12 and folate may also exist in other conditions, such as coronary artery disease and fracture risk (22, 23). Therefore, the probability that excess levels of vitamin B12 and folate may impair female fertility cannot be excluded by our findings and should be considered with caution in infertility clinics. Last, neither a beneficial association nor adverse association was revealed for pantothenic acid and vitamin B6 in our study, despite animal research, indicating that vitamin B6 may have a beneficial role in oocyte quality (14). However, given the lack of evidence, it is too soon to draw conclusions about these associations.

Cluster data from IVF-ET of informative cluster size (such as oocyte counts or number transferred) makes the traditional univariate model, logistic, or multiple linear regressions incompetent when performing statistical analysis (44). Thus, a GEE-Poisson model was adopted in this study to take full consideration of cluster sizes; this may reveal more accurate and robust results. Another strength of our study lies in the use of HPLC-MS/MS, the gold standard for the detection of most B vitamins (40). Unfortunately, thiamine and biotin were beyond the scope of this study because the detection panel was unable to test serum levels. Finally, the study population enrolled couples undergoing infertility treatment who took nutrient supplements, such as vitamin B complex; this, along with the small sample size and problem with multiple testing, may have weakened the strength of our study. Studies with larger sample size or/and focusing on fewer substances of interest are expected in future. Nevertheless, this research highlights the importance of the pre-conception B vitamin status in female fertility.

## Conclusion

After a comprehensive investigation of most B vitamins during IVF-ET, it is suggested that pre-conception serum riboflavin, folate, and vitamin B12 may affect early embryo development and clinical outcomes. These findings offer insights into the optimal nutritional conditions for pregnancy planning and infertility treatment, but more evidence is needed to confirm our conclusion.

## Data availability statement

The original contributions presented in the study are included in the article/Supplementary material, further inquiries can be directed to the corresponding authors.

## Ethics statement

The studies involving human participants were reviewed and approved by Ethics Committee of Zhejiang Provincial People's Hospital. The patients/participants provided their written informed consent to participate in this study.

## Author contributions

S-sL and ZJ: data curation. LingZ and S-sL: formal analysis. C-yX: methodology. LingZ, L-mW, and Y-qT: resources. X-lL: software. W-hX, X-lW, and JS: supervision. LingZ and LinZ: writing—original draft. JS: writing—review and editing. All authors read and approved the final manuscript.

## Funding

This study was supported by the General Research Program for Medicine and Health of Zhejiang Province (2022KY575 to X-lW and 2021KY065 to LingZ), New Century 151 Talent Program of Zhejiang Province (LingZ), and Adjunct Talent Fund of Zhejiang Provincial People's Hospital.

## Conflict of interest

Authors X-lL and C-yX were employed by Calibra Lab, DIAN Diagnostics. The remaining authors declare that the research was conducted in the absence of any commercial or financial relationships that could be construed as a potential conflict of interest.

## Publisher's note

All claims expressed in this article are solely those of the authors and do not necessarily represent those of their affiliated organizations, or those of the publisher, the editors and the reviewers. Any product that may be evaluated in this article, or claim that may be made by its manufacturer, is not guaranteed or endorsed by the publisher.
